# Inhibition of interleukin 6 signalling and renal function: A Mendelian randomization study

**DOI:** 10.1111/bcp.14725

**Published:** 2021-02-10

**Authors:** David K. Ryan, Ville Karhunen, Drew J. Walker, Dipender Gill

**Affiliations:** ^1^ Clinical Pharmacology Group, Pharmacy and Medicines Directorate St George's University Hospitals NHS Foundation Trust London UK; ^2^ Clinical Pharmacology and Therapeutics Section, Institute of Medical and Biomedical Education and Institute for Infection and Immunity, St George's University of London London UK; ^3^ Department of Epidemiology and Biostatistics, School of Public Health Imperial College London London UK; ^4^ Novo Nordisk Research Centre Oxford, Old Road Campus Oxford UK

**Keywords:** cardiovascular disease, chronic kidney disease, interleukin‐6, Mendelian randomization study

## Abstract

Inhibition of interleukin 6 (IL‐6) signalling has been proposed as a potential cardioprotective strategy for patients with chronic kidney disease (CKD), but the direct effects of IL‐6 inhibition on renal function are not known. A Mendelian randomization (MR) study was performed to investigate the association of genetically proxied inhibition of IL‐6 signalling with estimated glomerular filtration rate (eGFR), CKD and blood urea nitrogen (BUN). Inverse‐variance weighted MR was used as the main analysis, with sensitivity analyses performed using simple median, weighted median and MR‐Egger methods. There was no evidence for an association of genetically proxied inhibition of IL‐6 signalling (scaled per standard deviation unit decrease in C‐reactive protein) with log eGFR (0.001, 95% confidence interval −0.004‐0.007), BUN (0.009, 95% confidence interval −0.003‐0.021) and CKD (odds ratio 0.948, 95% confidence interval 0.822‐1.094). These findings do not raise concerns for IL‐6 signalling having large adverse effects on renal function.

**What is already known about this subject**

- Inhibition of interleukin 6 (IL‐6) signalling has shown promising potential for lowering cardiovascular risk.
- Patients with chronic kidney disease (CKD) have a high burden of cardiovascular disease, likely mediated in part due to a low‐grade and persistent inflammatory state.
- There is growing interest in using IL‐6 inhibitors to reduce cardiovascular risk in patients with CKD.

**What this study adds**

- This Mendelian randomization study did not identify evidence to support the association of genetically proxied inhibition of IL‐6 signalling with changes in renal function.
- Adverse effects on renal function directly related to IL‐6 inhibition are unlikely to limit this therapeutic strategy for reducing cardiovascular disease risk in patients with CKD.

## 1 INTRODUCTION

Cardiovascular disease (CVD) accounts for half of all deaths in end‐stage renal failure and the burden of CVD in chronic kidney disease (CKD) is not fully explained by traditional risk factors.^1^ This suggests that alternative pathways may be implicated in the disproportionately high CVD risk in patients with declining renal function.^2^ CKD is recognized as a low‐grade but persistent inflammatory state, with raised levels of inflammatory biomarkers such as interleukin‐1β (IL‐1β), interleukin‐6 (IL‐6) and C‐reactive protein (CRP).^3^ Inflammation plays a critical role in atherosclerosis and it is possible that the inflammatory milieu of CKD contributes to the excessive risk of CVD in CKD.^4^, ^5^ Inflammatory markers including high‐sensitive CRP and IL‐6 are predictive of cardiovascular events and IL‐6 levels are independent predictors of CVD and mortality in patients with CKD.^6^, ^7^ Pharmacological inhibition of IL‐1β by the monoclonal antibody canakinumab has reduced rates of major cardiovascular events in patients with CKD who had a previous myocardial infarction.^8^ Further analysis of canakinumab showed that the cardioprotective effect was dependent on inhibition of IL‐6 levels in a general population.^9^ There are now ongoing plans to commence trials of the IL‐6 signalling inhibitor ziltivekimab for reduction of CVD in patients with CKD.^10^ However, it has not been established whether or not direct IL‐6 signalling inhibition has an impact on renal function.

Mendelian randomization (MR) employs genetic polymorphisms as instrumental variables to study the effect of an exposure on an outcome.^11^ MR is less susceptible to confounding due to the random allocation of genetic variants and balancing of environmental factors at conception. For a valid MR study, the following assumptions must hold: the genetic proxy must be associated with the exposure, the genetic variant only affects the outcome through the exposure of interest with no horizontal pleiotropic effect and the genetic variant is not associated with any known confounder affecting the exposure and the outcome.^11^ A valid MR study is analogous to an endogenous randomized controlled trial based on the randomization of genetic variants at conception. Applied to drug development, MR provides an in silico platform to predict adverse drug consequences, explore drug repurposing and determine whether or not new therapeutic strategies are suitable to be trialled among vulnerable populations, such as patients with CKD.^12^ Considering the growing interest in IL‐6 inhibition in patients with CKD, the aim of the present study was to investigate the effect of inhibition of IL‐6 signalling on renal function by MR methods.

## 2 METHODS

A two‐sample MR study was conducted to investigate the association of genetically proxied inhibition of IL‐6 signalling with different measures of renal function: estimated glomerular filtration rate (eGFR), CKD and blood urea nitrogen (BUN). Two‐sample refers to the fact that the instrument‐exposure and instrument‐outcome estimate are obtained from two different genome‐wide association studies (GWAS), in this case serving to increase the statistical power of the MR study.

Genetic variants for downregulated IL‐6 signalling were selected as uncorrelated (*r* ^*2*^ < 0.1) single‐nucleotide polymorphism (SNPs) within 300 kB of the IL‐6 receptor gene (*IL6R,* GRCh37/hg19 coordinates: chr1:154077669‐154 741 926) that is associated with CRP in the UK Biobank^12^ (n = 337 199, White British ancestry individuals) at genome‐wide significance (*P* < 5E‐8). CRP is a reliable downstream marker for IL‐6 signalling and thus variants in the IL6R gene which associate with CRP levels represent proxies for IL‐6 signalling modulation. To further investigate the validity of the selected variants, we measured the Pearson coefficient for the correlation between their association with CRP and other markers of IL‐6 signalling, IL6R and serum IL‐6 levels, obtained from an independent GWAS.^13^ The variance in CRP levels explained by the genetic variants, *R* ^2^, was calculated using the formula: *R* ^2^ = [2 × MAF × (1 – MAF) × *β* ^2^], where MAF is the minor allele frequency and *β* is the effect estimate of the SNP on CRP levels. *F*‐statistics, a measure of instrument strength in MR, were calculated using the formula: *F* = *R* ^2^ × (n − 2)/(1 − *R* ^2^) where n is the number of individuals in the GWAS analysis.

Summary GWAS data from the Chronic Kidney Disease Genetics (CKDGen) Consortium European ancestry meta‐analyses were used to obtain genetic association estimates for the primary outcomes of log eGFR, BUN and CKD (Table 1).^14^ There are no overlapping populations between the exposure and outcome GWAS. In the original study, log eGFR was calculated using the Chronic Kidney Disease Epidemiology Collaboration (CKD‐EPI) equation in adults and using the Schwartz formula for participants who were 18 years or younger. CKD was constructed as a binary outcome based on an eGFR < 60 mL min^−1^ per 1.73 m^2^. BUN was calculated as 2.8 × blood urea (mg/dL). Power was calculated using an online tool (https://shiny.cnsgenomics.com/mRnd) to estimate the minimum and maximum effects that we had 80% statistical power to detect.

**TABLE 1:** Data sources for exposure and outcomes

Data	Data source	Population ancestry	Sample size	Exposure definition	Adjustments
Genetic variants in or near IL‐6R associated with variation in CRP	UK Biobank^12^	White British	337 199	Standard deviation change in CRP (4.35 mg/L) per copy increment of the effect allele	Age, sex and principal components of genetic ancestry
Blood urea nitrogen (BUN)	Chronic kidney disease genetics consortium meta‐analysis (n = 24 studies)^14^	European	243 029	Change in BUN (mg/dL) per copy increment of effect allele	Sex and age in all included studies, with some studies in the meta‐analysis further adjusting for study site, relatedness and principal components of genetic ancestry
Estimated glomerular filtration rate (eGFR)	Chronic kidney disease genetics consortium meta‐analysis (n = 42 studies)^14^	European	567 460	Change in log eGFR (mL min^−1^ per 1.73 m^2^) per copy increment of effect allele	
Chronic kidney disease Binary outcome eGFR > 60 mL min^−1^ per 1.73 m^2^	Chronic kidney disease genetics consortium meta‐analysis (n = 23 studies)^14^	European	480 698 (41 395 cases, 439 303 controls)	Log odds ratio for CKD per copy increment of effect allele	

Table 1 describes the source, population ancestry, sample size and exposure definitions for the genome‐wide association studies used in the present Mendelian randomization analysis.

Data for the exposure and outcome were harmonized according to the effect allele and no exclusions were made for palindromic variants. Individual MR estimates were calculated using the Wald ratio. Heterogeneity was assessed using Cochran's *Q* statistic and to account for heterogeneity a random‐effects inverse‐variance weighted method was used for the primary MR analysis. To explore potential pleiotropy, we conducted sensitivity analyses using the simple median, weighted median and MR‐Egger methods. The median methods are robust if less than 50% of the contribution to the MR estimates comes from invalid instrumental variables.^15^ MR‐Egger provides robust estimates even when all instrumental variables are invalid, as long as the INstrument Strength Independent of Direct Effect (INSIDE) assumption holds: that any pleiotropic effect of the variants on the outcome are independent of the strength of their association with the exposure.^15^ The estimated MR‐Egger intercept is indicative of the average pleiotropic effect of the variants used.^15^ We tested for such pleiotropy by assessing whether our intercept was significantly different from zero.^15^ Results are presented as effect estimates and corresponding 95% confidence intervals per standard deviation decrease in CRP levels. For eGFR and BUN, respectively, estimates represent the change in log eGFR or blood urea nitrogen, and for CKD the results are expressed as odds ratio for CKD. All data analyses were performed using “TwoSampleMR” package version 4.26 in R statistical software.

In further sensitivity analysis, we repeated our analysis using a different set of instrumental variables that have been used in a previous study to proxy IL‐6 signalling inhibition.^16^ These variants were selected based on associations with CRP (*P* < 5E‐8, clumped at *r* ^2^ < 0.1) in the Cohorts for Heart and Aging Research in Genomic Epidemiology (CHARGE) Inflammation Working Group GWAS of 204 402 individuals of European ancestry.^17^ Data from UK Biobank was chosen for the primary analysis because there were overlapping studies between exposure and outcome data sources.

## 3 RESULTS

Thirty SNPs were used as instrumental variables to represent genetically proxied inhibition of IL‐6 signalling (Table 2). The *F*‐statistic for the genetic exposure associations ranged between 40.28 and 1713.82 (median 98.0), indicating strong associations between the IL6‐R variants and CRP level (Table 2). The genetic association with CRP in the UK Biobank showed a high degree of correlation with other markers of IL‐6 signalling: IL6R (*r* = −0.90, *P* = 1.75E‐11) and serum IL‐6 levels (*r* = −0.80, *P* = 6.2E‐4, Appendix 1).

**TABLE 2:** Instrumental variables: variants employed as instrumental variables to proxy inhibition of IL‐6 signalling (associations with C‐reactive protein are detailed)

SNP	Effect allele	Other allele	Effect allele frequency	Beta	Standard error	*P* value	*R* ^2^	*F*
rs112505856	T	C	0.039	−0.046	0.006	8.82E‐13	1.58E‐04	74.48
rs16835819	C	T	0.018	−0.077	0.009	7.73E‐18	2.15E‐04	101.05
rs61806853	C	T	0.050	−0.044	0.006	2.26E‐15	1.79E‐04	84.22
rs79505546	T	C	0.017	−0.054	0.009	7.21E‐09	9.62E‐05	45.2
rs1194610	C	T	0.235	0.020	0.003	6.01E‐13	1.48E‐04	69.47
rs67156297	A	G	0.262	0.036	0.003	9.96E‐40	4.97E‐04	233.76
rs12077265	G	T	0.155	−0.056	0.003	6.24E‐64	8.20E‐04	385.54
rs4133213	A	C	0.450	−0.086	0.003	1.00E‐200	3.63E‐03	1713.82
rs79219014	T	G	0.028	−0.086	0.007	1.26E‐31	3.92E‐04	184.24
rs186110340	G	C	0.024	0.056	0.008	5.02E‐12	1.48E‐04	69.61
rs139952834	T	C	0.013	0.062	0.011	3.36E‐08	9.84E‐05	46.23
rs113580743	A	G	0.039	0.049	0.006	4.12E‐15	1.81E‐04	85.11
rs139460294	C	T	0.016	−0.059	0.010	4.48E‐09	1.06E‐04	49.83
rs140615642	C	T	0.020	−0.078	0.009	8.53E‐19	2.37E‐04	111.53
rs116059394	G	A	0.059	0.049	0.005	8.73E‐21	2.60E‐04	122.33
rs56100876	A	G	0.019	−0.109	0.009	2.35E‐33	4.41E‐04	207.24
rs4845645	A	T	0.173	−0.051	0.003	1.31E‐55	7.34E‐04	344.98
rs77994623	T	C	0.167	0.047	0.003	7.08E‐49	6.16E‐04	289.57
rs76289529	T	C	0.038	−0.047	0.006	8.22E‐14	1.64E‐04	76.86
rs12750774	A	G	0.316	−0.064	0.003	1.33E‐137	1.77E‐03	834.94
rs147483024	T	G	0.018	0.066	0.010	7.47E‐12	1.55E‐04	73.01
rs3766925	A	T	0.227	−0.016	0.003	4.12E‐08	8.57E‐05	40.28
rs12059682	C	T	0.206	0.046	0.003	5.39E‐55	6.97E‐04	327.51
rs188727323	T	C	0.189	−0.044	0.003	5.35E‐39	5.80E‐04	272.64
rs4845657	C	T	0.199	0.040	0.003	1.49E‐39	5.03E‐04	236.5
rs12757447	G	T	0.016	−0.063	0.010	1.43E‐10	1.23E‐04	58
rs79753070	A	G	0.025	−0.052	0.008	8.96E‐11	1.29E‐04	60.64
rs34693607	G	C	0.214	−0.033	0.003	2.09E‐29	3.62E‐04	170.15
rs11264245	T	C	0.057	−0.029	0.005	3.12E‐08	8.78E‐05	41.25
rs7523010	A	T	0.213	0.025	0.003	2.13E‐15	2.02E‐04	94.96

Table 2 shows the summary data for the variants that proxy IL‐6 signalling inhibition. SNP, single‐nucleotide polymorphism. Beta is the standard deviation unit change in CRP (4.35 mg/L) per copy increment in the effect allele. *R* ^2^ represents the variance in CRP explained by the respective genetic variant. The *F*‐statistic measures the strength of the instrumental variable with the exposure. In an additive model assuming independent variants, these instrumental variables explain 0.0138 of the variance in the exposure.

**FIGURE 1:**
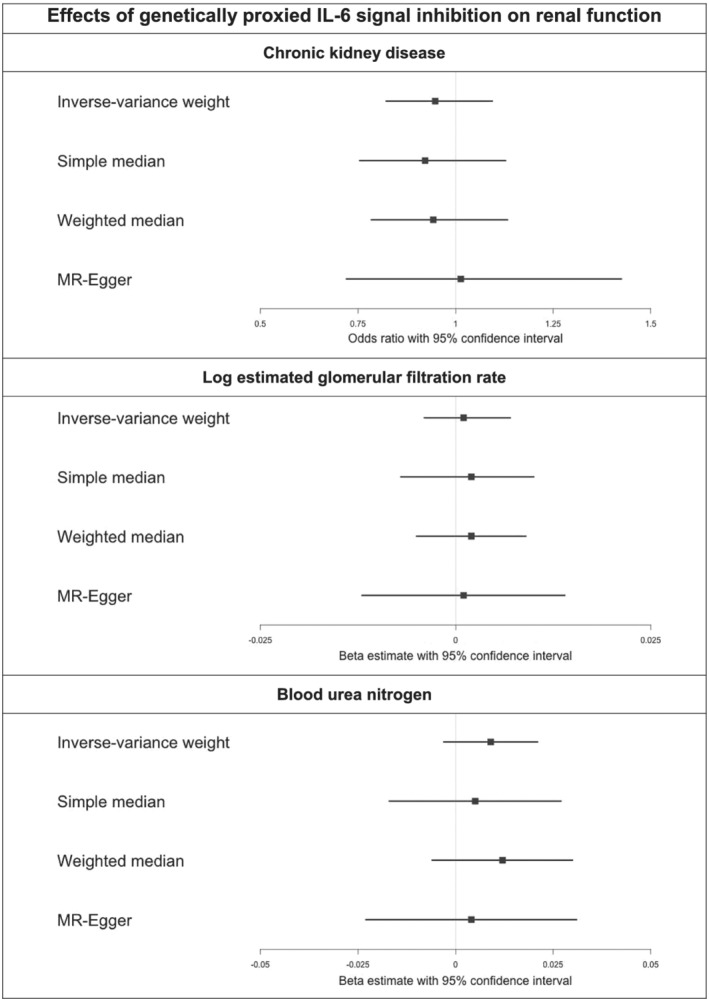
Effects of genetically proxied IL‐6 signal inhibition on renal function

In the main analysis, there was no strong evidence for an association of genetically proxied inhibition of IL‐6 inhibition with log eGFR (0.001, 95% confidence interval −0.004‐0.007), BUN (0.009, 95% confidence interval −0.003‐0.021) and CKD (odds ratio 0.948, 95% confidence interval 0.822‐1.094). The results were consistent across all considered measures of renal function (Figure 1). There was evidence of heterogeneity in the main MR analyses for eGFR and CKD, but no heterogeneity for BUN (Appendix 2). The MR‐Egger intercepts did not identify evidence of pleiotropy for eGFR, BUN or CKD (*P* = 0.912, *P* = 0.798 and *P* = 0.681, respectively). Individual SNP associations are provided in Appendix 3. Similar results were obtained in sensitivity analyses using instrumental variables obtained from the CHARGE consortium (Appendix 5).

## 4 DISCUSSION

This MR study did not identify evidence to show that genetically proxied inhibition of IL‐6 signalling has an effect on renal function. IL‐6 inhibitors such as tocilizumab are currently licenced for use in rheumatoid arthritis, juvenile arthritis and more recently have been under investigation for treatment of excessive inflammation in patients with severe acute respiratory syndrome coronavirus 2 infection.^18^ Atherosclerotic cardiovascular disease is an inflammatory disorder and both MR studies and randomized controlled trials suggest that inhibition of IL‐6 signalling reduces risk of cardiovascular outcomes and thromboembolic events.^16^, ^19^ Given the disproportionate burden of cardiovascular disease in patients with CKD and the inflammatory nature of both these conditions, there is growing interest in repurposing IL‐6 inhibitors to treat CVD in CKD.^10^ Our current findings support pharmacological IL‐6 inhibition being unlikely to have a direct adverse effect on renal function.

The findings of this MR study are in line with an earlier study of renal function in patients with rheumatoid arthritis and renal insufficiency receiving tocilizumab therapy.^20^ However, this observational study is small (120 participants), had large numbers of patient stopping or switching therapy (60% switching biological therapy) and may be susceptible to confounding factors (patients receiving IL‐6 inhibition were older and had rheumatoid arthritis for longer).^20^ More recently, the Il‐1β inhibitor canakinumab, which also inhibits IL‐6, was trialled in patients with CKD and showed no effect on renal function in terms of serial eGFR, creatinine and urinary albumin‐creatinine ratio.^8^ The present MR study adds support to the initial pharmacovigilance surveys, and further is less prone to confounding and reverse causation. The manufacturer of tocilizumab (tradename Actemra) advises that no dose adjustment is required for patients with mild renal impairment, but cautions that the drug has not been studied in patients with moderate to severe renal dysfunction.^21^ This highlights a potential limitation of available clinical trial data: despite over 10% of patients in the developed world having renal impairment, patients with CKD are excluded from up to 75% of all randomized‐controlled trials.^22^ MR may help in evaluating the safety of drugs in silico prior to trials in patients. For example, MR drug safety studies have in the past substantiated the causal relationship between inhibition of IL‐6 signalling and increased risk of pneumonia.^18^ Furthermore, MR can provide more immediate drug safety information compared to usual pharmacovigilance strategies, such as the Medicines and Healthcare Products Regulatory Agency yellow card scheme.

Our study has a number of strengths. In an attempt to minimize the bias related to pleiotropic effects of variants, instrumental variables were selected based on their proximity to the *IL6R* gene and in relation to reliable biomarkers of IL‐6 signalling. In addition, the MR‐Egger method did not provide evidence to suggest biasing pleiotropy. Our results were robust to sensitivity analysis where different SNPs to proxy IL‐6 signalling inhibition were selected from an independent GWAS. Furthermore, the consistency of our results across different MR methods and different measurements of renal dysfunction further substantiates the null findings.

Our study also has limitations. The analysis for eGFR was well‐powered and it is unlikely that the null finding for eGFR represents a type II error for a clinically relevant effect. There was less power to detect small differences for BUN and CKD due to the smaller number of participants or cases, respectively, in the GWAS. It is important to interpret our findings within the context of an MR study, which considers genetically proxied inhibition of IL‐6 signalling, rather than the effect of a discrete clinical intervention. Our approach looks at IL‐6 signalling in isolation, and it is possible that pharmacological IL‐6 inhibitors could have off‐target effects (aside from IL‐6R signalling) on other renal or extrarenal pathways which may ameliorate or exacerbate renal function indirectly. There is also the possibility of drug‐drug interactions that cannot be accounted for in the present MR analysis.

In conclusion, this study is consistent with the hypothesis that inhibition of IL‐6 signalling does not directly affect renal function, supporting this approach as a therapeutic opportunity for reducing the risk of CVD in patients with CKD.

## COMPETING INTERESTS

D.G. is employed part‐time by Novo Nordisk. The remaining authors have no conflicts of interest to declare.

## CONTRIBUTORS

D.K.R. conducted analysis and drafted manuscript. V.K. conducted analysis, contributed to statistical analysis and manuscript development. D.J.W. verified analysis, contributed to the manuscript and provided clinical context to study. D.G. conceived study, verified analysis and edited manuscript. All authors reviewed and edited manuscript.

## Figures and Tables

**FIGURE A1:**
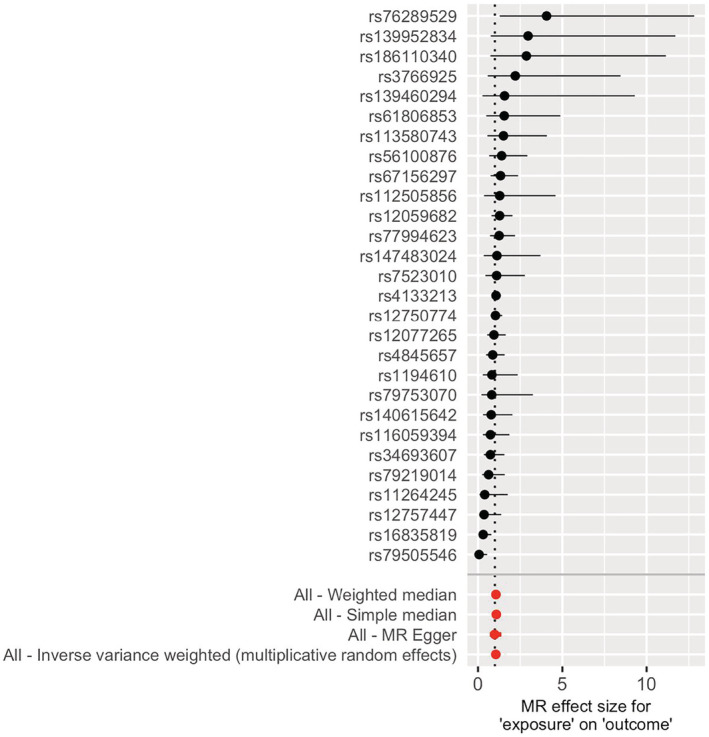
Chronic kidney disease: associations of genetically proxied IL‐6 inhibition on chronic kidney disease for each instrumental variable. Forest plot showing the individual Wald ratios and 95% confidence intervals for each variant used in the primary analysis. Pooled estimates using inverse‐variance weighted, simple median, weighted median and MR‐Egger are shown in red

**FIGURE A2:**
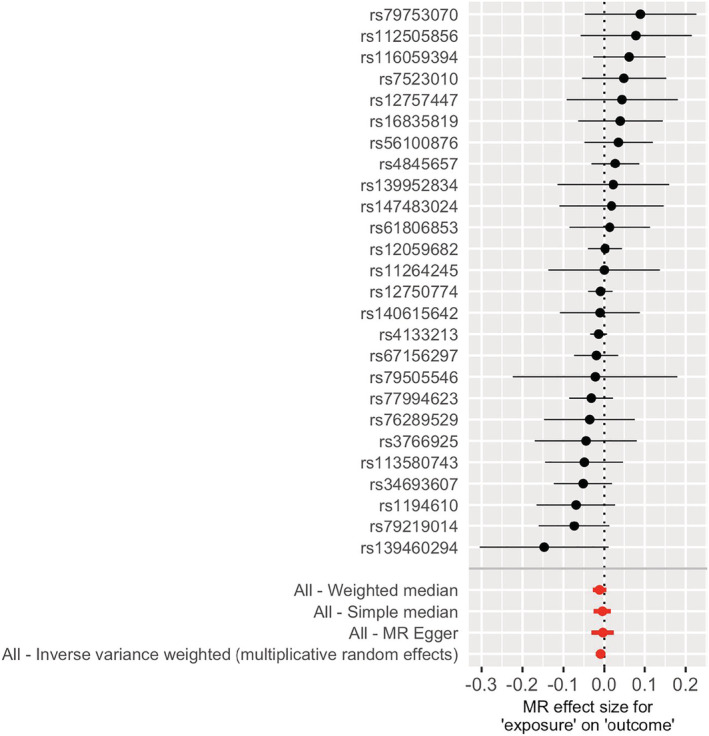
Blood urea nitrogen: associations of genetically proxied IL‐6 inhibition on blood urea nitrogen for each instrumental variable. Forest plot showing the individual Wald ratios with 95% confidence intervals for each variant used in the primary analysis. Pooled estimates using inverse‐variance weighted, simple median, weighted median and MR‐Egger are shown in red

**FIGURE A3:**
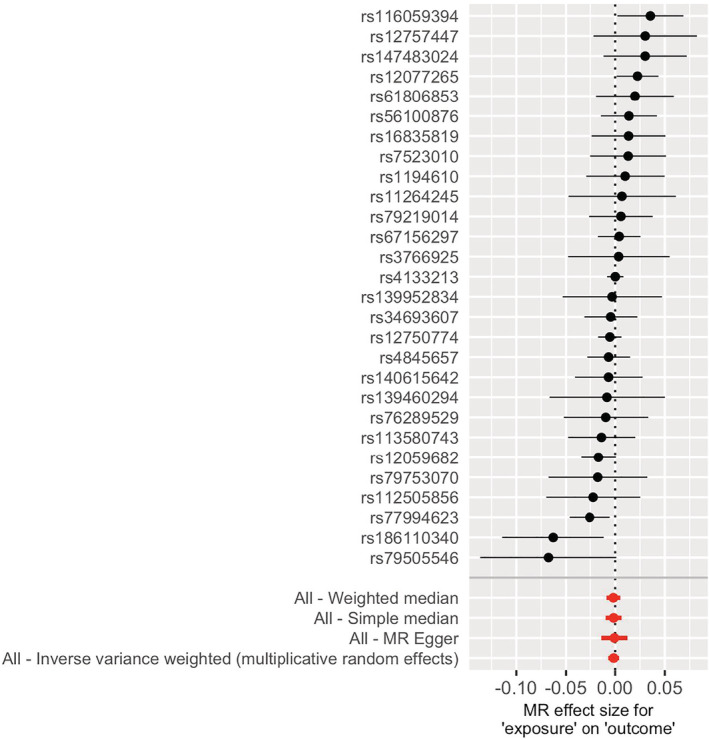
Log estimated glomerular filtration rate (eGFR): associations of genetically proxied IL‐6 inhibition on eGFR for each instrumental variable. Forest plot showing the individual Wald ratios with 95% confidence intervals for each variant used in the primary analysis. Pooled estimates using inverse‐variance weighted, simple median, weighted median and MR‐Egger are shown in red

## Data Availability

All data used in this study are publicly available. The statistical code used in this work is available from the corresponding author upon reasonable request.

## APPENDIX 1 Genetic association estimates for variants proxying the effect of IL‐6 inhibition

Genetic proxies for interleukin 6 (IL‐6) inhibition selected on basis of their association with C‐reactive protein (CRP), and the corresponding associations with soluble interleukin 6 receptor and serum IL‐6

**Table:**

SNP	Effect allele	Other allele	Beta estimate for association with CRP (UK Biobank)	Beta estimate for association with IL6R (INTERVAL study)	Beta estimate for association with serum IL‐6 (INTERVAL study)
rs112505856	T	C	−0.046	0.316	
rs16835819	C	T	−0.077	0.453	
rs61806853	C	T	−0.044	0.496	
rs79505546	T	C	−0.054	0.356	
rs1194610	C	T	0.020	0.190	
rs67156297	A	G	0.036	−0.254	−0.254
rs12077265	G	T	−0.056	−0.075	−0.044
rs4133213	A	C	−0.086	0.974	0.084
rs79219014	T	G	−0.086	0.758	0.087
rs186110340	G	C	0.056	−0.548	
rs139952834	T	C	0.062	−0.651	
rs113580743	A	G	0.049	−0.514	
rs139460294	C	T	−0.058	0.384	0.136
rs140615642	C	T	−0.078	0.547	
rs116059394	G	A	0.048	−0.576	−0.064
rs56100876	A	G	−0.109	0.584	
rs4845645	A	T	−0.051	0.219	0.082
rs77994623	T	C	0.047	−0.618	
rs76289529	T	C	−0.047	0.666	0.107
rs12750774	A	G	−0.064	0.549	0.057
rs147483024	T	G	0.066	−0.308	−0.156
rs3766925	A	T	−0.016	0.184	−0.049
rs12059682	C	T	0.046	−0.383	−0.055
rs188727323	T	C	−0.043		
rs4845657	C	T	0.040	−0.273	
rs12757447	G	T	−0.063	0.253	
rs79753070	A	G	−0.052	0.571	0.142
rs34693607	G	C	−0.033	0.196	0.058
rs11264245	T	C	−0.029	0.447	
rs7523010	A	T	0.025	−0.049	

Pearson correlations:

- Correlation between CRP and IL6R association: *r* = −0.90, *P* = 1.8E‐11
- Correlation between CRP and IL‐6 association: *r* = −0.80, *P* = 6.2E‐4

Table showing the association between SNPs and CRP, IL‐6 receptor and serum IL‐6 level. SNP, single nucleotide polymorphism. Beta is the standard deviation unit change in CRP, IL‐6 alpha subunit or IL‐6 level per copy increment in the effect allele. The association between individual variants and CRP was established in the UK Biobank. Associations between individual variants and IL‐6 receptor and serum IL‐6 were obtained from the INTERVAL study^13^ as made publicly available through the PhenoScanner database.^23^ Missing cells infer no data was available for the respective variant in the INTERVAL study.

## APPENDIX 2 Mendelian randomization sensitivity analysis estimates for the association of genetically proxied inhibition of IL‐6 signalling with measures of renal function

**TABLE A1:** Chronic kidney disease: effects of genetically proxied IL‐6 inhibition on chronic kidney disease

Method	Estimate (standard error)	Odds ratio (95% confidence interval)	*P* value
Inverse‐variance weighted	−0.053 (0.073)	0.948 (0.822‐1.094)	0.474
Simple median	−0.081 (0.103)	0.922 (0.754‐1.128)	0.431
Weighted median	−0.059 (0.094)	0.943 (0.784‐1.133)	0.528
MR‐Egger	0.013 (0.174)	1.013 (0.720‐1.425)	0.942
MR‐Egger intercept	−0.004 (0.174)	0.996 (0.708‐1.401)	0.681

Cochran's Q‐statistic: inverse‐variance weighted analysis 35.38 (SNPs 27), *P* = 0.13.

**TABLE A2:** Log estimated glomerular filtration rate: association of genetically proxied inhibition of IL‐6 signalling with log odds of estimated glomerular filtration rate

Method	Estimate (standard error)	95% confidence interval	P value
Inverse‐variance weighted	0.001 (0.003)	−0.004‐0.007	0.599
Simple median	0.002 (0.004)	−0.007‐0.010	0.721
Weighted median	0.002 (0.004)	−0.005‐0.009	0.620
MR‐Egger	0.001 (0.007)	−0.012‐0.014	0.908
MR‐Egger intercept	0.0004 (0.0003)	−0.0006‐0.0007	0.912

Cochran's Q‐statistic: inverse‐variance weighted analysis 37.93 (SNPs 27), *P* = 0.08.

**TABLE A3:** Blood urea nitrogen: effects of genetically proxied IL‐6 inhibition on blood urea nitrogen

Method	Estimate (standard error)	95% confidence interval	*P* value
Inverse‐variance weighted	0.009 (0.006)	−0.003‐0.021	0.121
Simple median	0.005 (0.011)	−0.017‐0.027	0.666
Weighted median	0.012 (0.009)	−0.006‐0.030	0.183
MR‐Egger	0.004 (0.014)	−0.023‐0.031	0.798
MR‐Egger intercept	0.0003 (0.0007)	−0.0016‐0.001	0.662

Cochran's Q‐statistic: inverse‐variance weighted analysis 22.24 (SNPs 25), *P* = 0.62.

## APPENDIX 4 Power calculations for the primary Mendelian randomization analyses

Table detailing the minimum and maximum estimates that the primary analysis had 80% statistical power to detect. Power calculation were based on the online application: https://shiny.cnsgenomics.com/mRnd. The total variance explained by the variants was estimated using their total *R* ^2^.

**Table:**

Continuous outcomes		
Phenotype	n total	Detectable MR‐estimate at 80% power
Log eGFR	567 460	≤−0.0000405, ≥0.000034
BUN	243 029	≤−0.0158, ≥0.0157

Categorical outcomes		
Phenotype	n total (% cases)	Detectable OR at 80% power
CKD	480 698 (8.6%)	≤0.879 or ≥1.124

## APPENDIX 5 Sensitivity analysis using variants from the CHARGE Consortium

Instrumental variables from the CHARGE Consortium: variants employed as instrumental variables to proxy inhibition of IL‐6 signalling. Associations with C‐reactive protein are detailed. Instruments (n = 7) were selected by based on the association of variants within 300kB of the IL6R gene with CRP in the CHARGE GWAS. These were originally identified by Georgakis et al.^16^

**Table:**

SNP	Effect allele	Other allele	Effect allele frequency	Beta	Standard error	*P* value	*R* ^2^	*F*
rs73026617	T	C	0.097	0.0474	0.0068	3.16E‐12	3.58E‐04	73.16
rs12083537	A	G	0.193	0.0643	0.0053	7.14E‐34	1.17E‐03	239.60
rs4556348	T	C	0.148	0.0541	0.0067	6.77E‐16	6.71E‐04	137.25
rs2228145	A	C	0.36	0.0899	0.0042	1.21E‐101	3.39E‐03	694.37
rs11264224	A	C	0.193	0.0465	0.0057	3.41E‐16	6.12E‐04	125.23
rs12059682	T	C	0.196	−0.0441	0.0049	2.26E‐19	5.57E‐04	113.96
rs34693607	C	G	0.184	0.0368	0.0057	1.07E‐10	3.70E‐04	75.59

Table showing the summary data for the variants that proxy IL‐6 signalling inhibition. SNP, single nucleotide polymorphism. Beta is the unit change in natural log transformed CRP (mg/L) per copy increment in the effect allele. *R* ^2^ represents the variance in CRP explained by the respective genetic variant. The *F*‐statistic measures the strength of the instrumental variable with the exposure. Variants identified and associated well with IL‐6 signalling inhibition in a previous Mendelian randomization study exploring IL‐6 inhibition and effects on ischaemic stroke and cardiovascular outcomes.^16^

## APPENDIX 6 Estimates for the association of genetically proxied inhibition of IL‐6 signalling with measures of renal function in sensitivity analysis

**TABLE A4:** Chronic kidney disease: association of genetically proxied IL‐6 inhibition on chronic kidney disease

Method	Estimate (standard error)	Odds ratio (95% confidence interval)	*P* value
Inverse‐variance weighted	−0.018 (0.052)	0.982 (0.887‐1.087)	0.723
Simple median	−0.009 (0.111)	0.991 (0.797‐1.232)	0.936
Weighted median	−0.006 (0.088)	0.994 (0.837‐1.181)	0.943
MR‐Egger	0.105 (0.239)	1.11 (0.695‐1.774)	0.678
MR‐Egger intercept	−0.008 (0.011)	0.992 (0.971‐1.013)	0.490

Cochran's Q statistic: Inverse‐variance weighted analysis: 2.96 (SNPs 6), *P* = 0.814.

**TABLE A5:** Log estimated glomerular filtration rate: association of genetically proxied inhibition of IL‐6 signalling with log odds of estimated glomerular filtration rate

Method	Estimate (standard error)	95% confidence interval	*P* value
Inverse‐variance weighted	0.001 (0.004)	−0.007‐0.009	0.862
Simple median	−0.004 (0.005)	−0.014‐0.006	0.376
Weighted median	−0.001 (0.003)	−0.007‐0.005	0.688
MR‐Egger	−0.006 (0.013)	−0.031‐0.019	0.687
MR‐Egger intercept	0.0004 (0.0008)	−0.002‐0.001	0.636

Cochran's Q statistic: Inverse‐variance weighted analysis 11.98 (SNPs 5), *P* = 0.062.

**TABLE A6:** Blood urea nitrogen: association of genetically proxied IL‐6 inhibition on blood urea nitrogen

Method	Estimate (standard error)	95% confidence interval	*P* value
Inverse‐variance weighted	0.008 (0.007)	−0.006‐0.022	0.218
Simple median	0.003 (0.010)	−0.017‐0.023	0.759
Weighted median	0.008 (0.009)	−0.01‐0.026	0.350
MR‐Egger	0.029 (0.023)	−0.016‐0.074	0.260
MR‐Egger intercept	−0.001 (0.001)	−0.003‐0.001	0.353

Cochran's Q statistic: inverse‐variance weighted analysis 5.30 (SNPs 5), *P* = 0.505

**Table:**

Sensitivity analysis: variants from the CHARGE consortium
Effects of genetically proxied IL‐6 signal inhibition on renal function
Chronic kidney disease

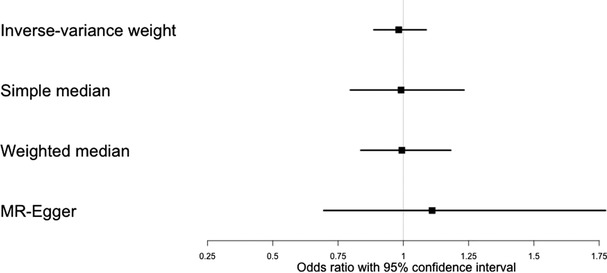

Log estimated glomerular filtration rate

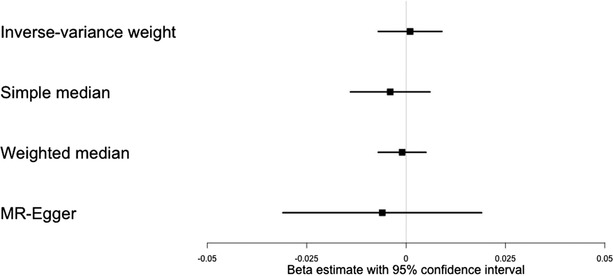

Blood urea nitrogen

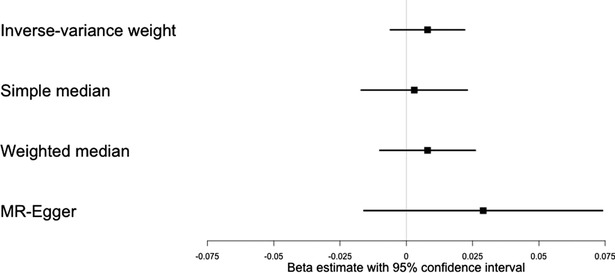

Forest plots showing the Mendelian randomization estimates for chronic kidney disease (CKD), log eGFR (estimated glomerular filtration rate) and blood urea nitrogen (BUN) with different analysis methods (simple median, weighted median, inverse‐variance weighted, MR‐Egger). eGFR and BUN are presented as causal estimates with 95% confidence interval. CKD is described as the odds ratio (95% confidence interval) of having CKD with genetically proxied IL‐6 signalling inhibition.
